# Statement of Retraction: B7 family member H4 induces epithelial-mesenchymal transition and promotes the proliferation, migration and invasion of colorectal cancer cells

**DOI:** 10.1080/21655979.2026.2745790

**Published:** 2026-11-05

We, the Editors and Publisher of the journal *Bioengineered*, have retracted the following article from publication.

Yin, Y., Shi, L., Yang, J., Wang, H., Yang, H., & Wang, Q. (2022). B7 family member H4 induces epithelial-mesenchymal transition and promotes the proliferation, migration and invasion of colorectal cancer cells. *Bioengineered*, 13(1), 107–118. https://doi.org/10.1080/21655979.2021.2009411

Since publication, significant concerns have been raised by a third party about the integrity of the data and reported results in the article.

When approached for an explanation, the authors have provided their original data and answered our queries, but these do not sufficiently address the concerns.

As verifying the validity of published work is core to the integrity of the scholarly record, we are therefore retracting the article. The authors have been informed of this decision. The author(s) do not agree with the retraction.

We have been informed in our decision-making by our policy on publishing ethics and integrity and COPE guidelines.

The retracted article will remain online to maintain the scholarly record and will be digitally watermarked on each page as ‘Retracted’.

